# An Improved Method for Growing Primary Neurons on Electron Microscopy Grids Co-Cultured with Astrocytes

**DOI:** 10.3390/ijms242015191

**Published:** 2023-10-14

**Authors:** Ishika Kumar, Anju Paudyal, Anna Kádková, Michelle Stewart, Jakob Balslev Sørensen, Julika Radecke

**Affiliations:** 1 Diamond Light Source, Harwell Science and Innovation Campus, Fermi Ave, Didcot OX11 0DE, UK; 12ishikakumar@gmail.com; 2 Mary Lyon Centre at MRC Harwell, Harwell Campus, Becquerel Ave, Didcot OX11 ORD, UK; a.paudyal@har.mrc.ac.uk (A.P.); m.stewart@har.mrc.ac.uk (M.S.); 3 Department of Neuroscience, University of Copenhagen, Blegdamsvej 3B, DK-2200 Copenhagen, Denmarkjakobbs@sund.ku.dk (J.B.S.)

**Keywords:** primary neurons, astrocytes, cryo-electron microscopy, synapse

## Abstract

With the increasing popularity of cryo-electron tomography (cryo-ET) in recent years, the quest to establish a method for growing primary neurons directly on electron microscopy grids (EM grids) has been ongoing. Here we describe a straightforward way to establish a mature neuronal network on EM grids, which includes formation of synaptic contacts. These synapses were thin enough to allow for direct visualization of small filaments such as SNARE proteins tethering the synaptic vesicle (SV) to the active zone plasma membrane on a Titan Krios without prior focused ion-beam milling.

## 1. Introduction

The synapse as a functional unit of pre- and postsynapse is the neuron’s main means of chemical communication with other neurons and target cells (such as muscle cells) outside the central nervous system. By releasing neurotransmitters stored in synaptic vesicles from the presynapse, which are detected by the postsynapse, signals are transmitted from one cell to the other. Alterations caused by de novo mutations of the synaptic release machinery can lead to neurodevelopmental disease within the first year of life [1], whereas synaptic defects accumulating over a lifetime can lead to neurodegenerative diseases [2]. The attractive possibility of studying healthy and diseased synapses by means of cryo-electron tomography (cryo-ET), which provides close-to-native structural resolution, has been discussed in a recent review [3].

Cryo-ET is ideally suited to studying the synapse at high structural resolution, e.g., for visualization of SNARE proteins or receptors in a close-to-native state. Similarly to [4], where a 10 Å resolution of spike proteins expressed on cells could be reached with enough data, macromolecules in the thinner regions of neuronal processes could be visualized via cryo-ET and subtomogram averaging [5]. In recent years, various papers have been published either on methods for growing neurons on electron microscopy (EM) support film (grids) [6,7,8] or analysing specific regions in neurons via cryo-ET [9,10]. However, for imaging functional synapses, the main challenge is obtaining either enough synapses [10] or finding them in sufficiently thin regions [7,9]. Previous methods papers [6,8] did not focus on obtaining functional, naturally formed synapses. Here, we introduce a simple protocol for growing small amounts of primary neurons on EM grids, which form mature neurons with many thin processes and functional synapses ideal for direct imaging in cryo-ET without prior thinning via focused-ion beam (FIB) milling.

Initially, when neurons were first being studied via cryo-ET, it was either in the form of cryo-sections of organotypic rat hippocampal slices or as synaptosomes (pinched-off nerve endings) [11]. While cryo-sections are difficult to obtain and suffer from sectioning artifacts [11], synaptosomes, while they can be very useful for certain studies such as time-resolved synaptic vesicle exocytosis [12], are not ideal for studying mutations in a rescue setting (expression in knockout (KO) neurons). Primary neurons grown directly on EM grids would be advantageous in, for example, studying SNARE mutations [12,13].

In recent years, a few papers have been published on how to grow neurons on EM grids [6,7] and how to grow neurons with artificial synapses [8], as well as some papers that were studying synapses by cryo-ET in neurons grown on EM grids [9,10,13]. The common feature that most of the papers in the cryo-EM field share is that an astrocyte feeder layer is absent [6,7,8,9], whereas others have used an astrocyte feeder layer, but the desired effect of obtaining more mature synapses in thin regions was not achieved [10,13]. On the other hand, papers outside the cryo-EM field that measure synaptic function routinely use astrocyte–neuron co-cultures, or astrocyte–neuron–microglia triple cultures, which improves neuron growth and synaptic function [14,15,16,17,18,19]. Astrocytes provide trophic support for neurons and are important for their proper phenotypic differentiation and maturation. Especially in lower cell density conditions, paracrine trophic support, either from surrounding neurons or astrocytes, is important for longer-term growth of neurons [14]. While media exists to support neuron growth without an astrocyte feeder layer, phenotypic differentiation of neurons grown without an astrocyte feeder layer has not been well characterised [14]. Thus, an astrocytic co-culture system for cryo-EM could be advantageous, as it would provide better trophic support for low-density neuron growth. At the same time, a low-density neuron culture would be immensely advantageous for cryo-EM with very few neuronal cell bodies obscuring the view of an EM grid but still having the trophic support to sprout extensive neuronal networks. Because astrocytes enable a longer-term growth of neurons, this means neurons could be grown into more mature states where we would expect more functional synapses. This is the ideal scenario for cryo-EM studies without the need for FIB milling, while at the same time obtaining neurons that develop into a closer-to-natural state.

Another common feature of neurons grown on EM grids has been the coating of the grid with poly-D/L-lysine (PDL/PLL) [6,7,8,9,10], which can be added to potentially improve cell distribution and spreading [20]. However, adding PDL/PLL adds another handling step for the grids as well as being a time-consuming preparation procedure which can be omitted with the method we propose here, which was used in a recent research article [12].

It should also be noted that growing neurons or cells in general on EM grids requires the use of the more fragile gold grids in contrast to the standard copper grids which are toxic for cells and are used commonly for samples in solution.

The method described here reduces handling steps by eliminating direct coating of the grid with PDL/PLL while adding an astrocytic feeder layer to improve neuronal growth and synaptic maturation. Additionally, as a proof of concept, the method has been performed by two different experimenters (Julika Radecke, JR (experimenter 1) and Ishika Kumar, IK (experimenter 2)) at two different institutes in different countries (JR performed experiments at Copenhagen University, while IK performed experiments at the electron Bio-Imaging Centre (eBIC) at Diamond Light Source and the Mary Lyon Mouse Facility).

## 2. Results

**Figure 1:**
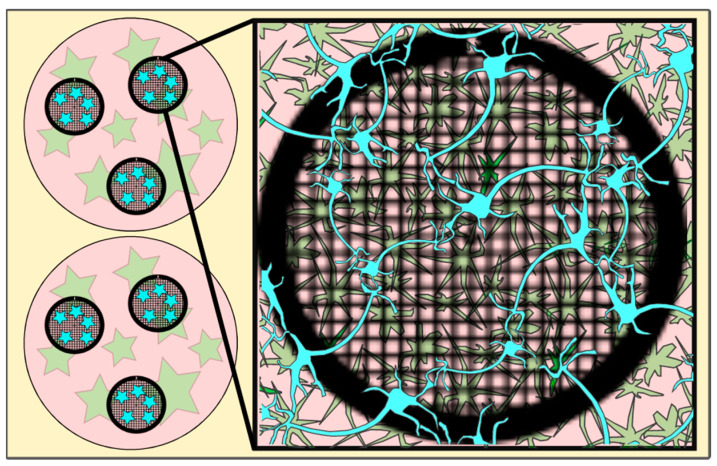
Cartoon depicting EM grids (black circles, meshed) in a well (pink circles) with astrocytes (green stars) grown on the well bottom and neurons (blue (stars)) incubated on the EM grid (for details, see Section 4) and after a 30-min incubation period transferred into the wells containing the astrocytes. Figure 1 has been modified from [12].

As part of a method development paper, the majority of the results are also the method and therefore can be found in Section 4. To ease understanding on how and where the neurons have been grown in relation to astrocytes, a cartoon depicting part of a well plate with astrocytes grown at the bottom of each well and an EM grid containing the neurons placed on top of the astrocyte culture is shown in Figure 1.

**Figure 2:**
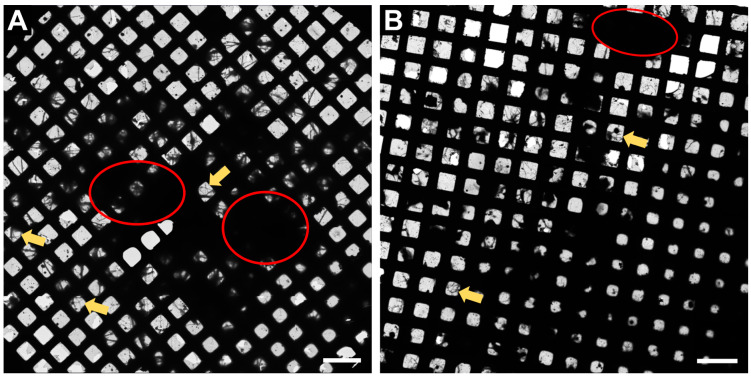
Representative Atlas images of neurons grown on 200 mesh (=200 gridsquares) gold grids. (**A**) Atlas images acquired at the core facility for integrated microscopy (CFIM) in Copenhagen by JR. (**B**) Atlas images acquired at eBIC by IK. Yellow arrows show few examples of neuronal processes crossing over a gridsquare. Red circles indicate regions of densely packed neurons. Scale bars = 200 µm.

**Figure 3:**
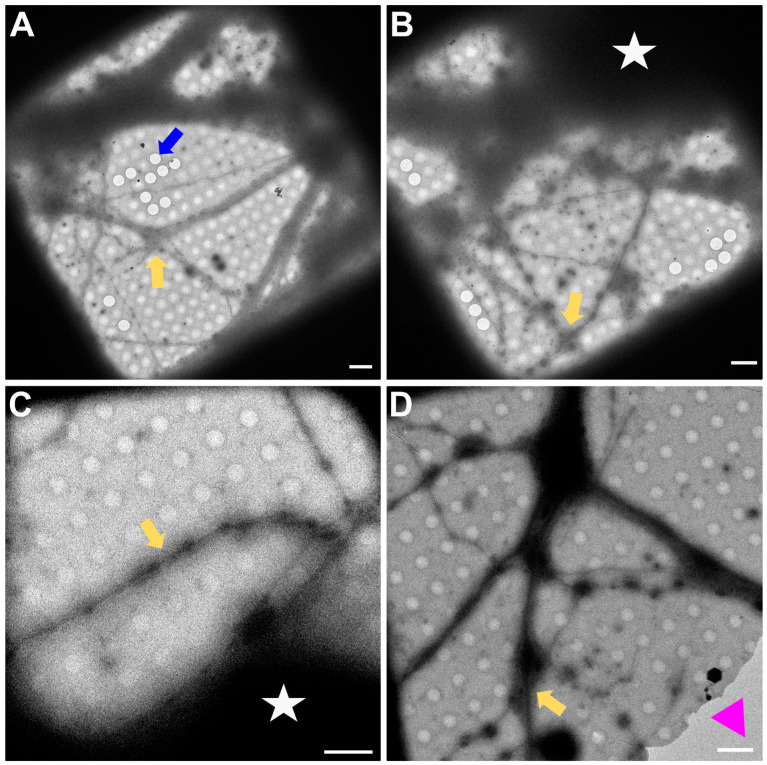
Gridsquare overview. (**A**,**B**) Overview images acquired at the CFIM by JR. Blue arrow pointing to an example of an empty hole. Yellow arrows point towards a neuronal crossing. White asterisk indicates a cell body. (**C**,**D**) Overview images acquired at eBIC by IK. Yellow arrows point towards a neuronal crossing. White asterisk indicates a cell body. Pink triangle is localized inside a broken part of carbon film. Scale bars = 5 µm. Figure (**A**) has been modified from [12].

**Figure 4:**
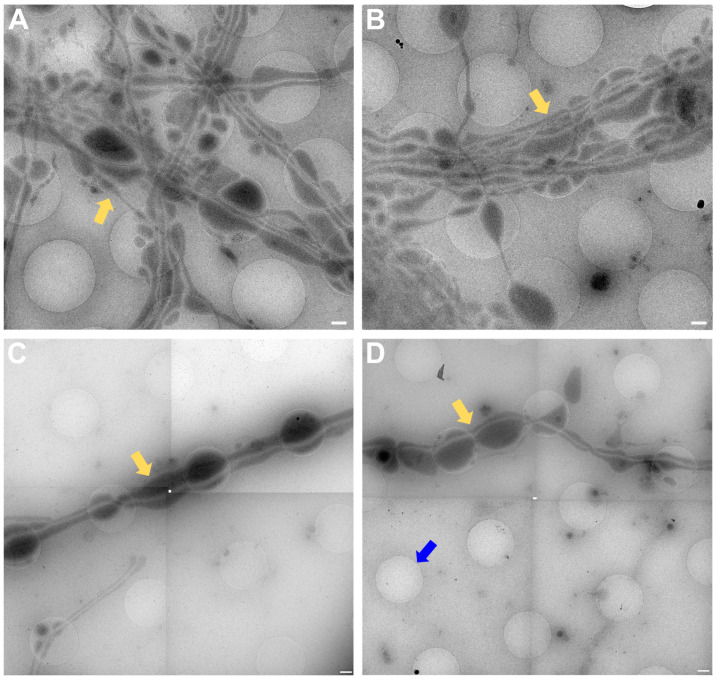
Search magnification. (**A**,**B**) Images acquired at the CFIM by JR. Yellow arrows point towards examples of neuronal processes. (**C**,**D**) Search maps acquired at eBIC by IK. Yellow arrows point towards examples of neuronal processes. The blue arrow points towards a foilhole example which is filled. Scale bars = 500 nm. Figure (**A**) has been modified from [12].

**Figure 5:**
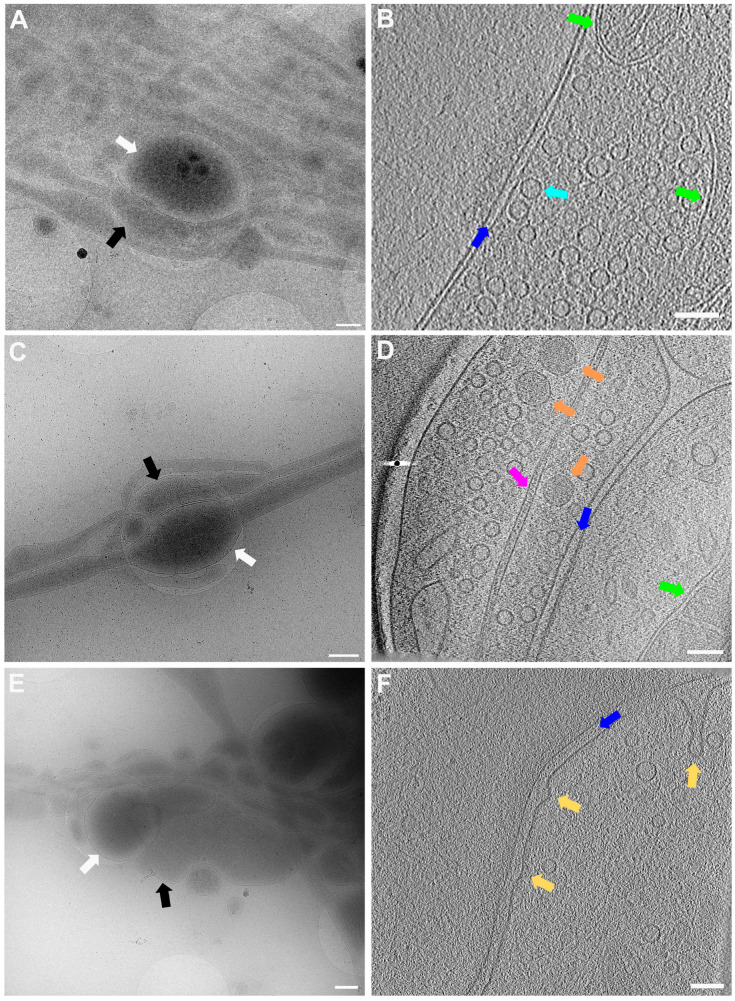
Lower and higher magnification images of a functional synapse constituting a pre- and a postsynapse. (**A**,**C**,**E**) Low magnification views of the regions where the synapses are located. Scale bars = 500 nm. (**B**) Corresponding tomogram to (**A**). Single slice of a reconstructed tomogram (acquired at 3.7 Å/px, reconstructed at binning 3; synapse thickness = 293 nm). Tomogram acquired at the CFIM by JR. Scale bar = 100 nm. Panel (**B**) has been modified from [12]. (**D**) Corresponding tomogram to (**C**). Single slice of a reconstructed tomogram (acquired at 2.9 Å/px, reconstructed at binning 4; synapse thickness = 200 nm). Full tomographic reconstruction in Supplementary Video S1. (**F**) Corresponding tomogram to (**E**). Ten slices averaged of a reconstructed tomogram (acquired at 2.9 Å/px, reconstructed at binning 4; synapse thickness = 330 nm). Full tomographic reconstruction in Supplementary Video S2. (**C**–**F**) Acquired at eBIC by IK. Scale bars for (**D**,**F**) = 100 nm. Labels: white arrows: postsynapse; black: presynapse; green: mitochondria; dark blue: synaptic cleft; light blue: synaptic vesicle; orange: large dense core vesicle; pink: microtubule; yellow: fusion and/or endocytosis events.

**Figure 6:**
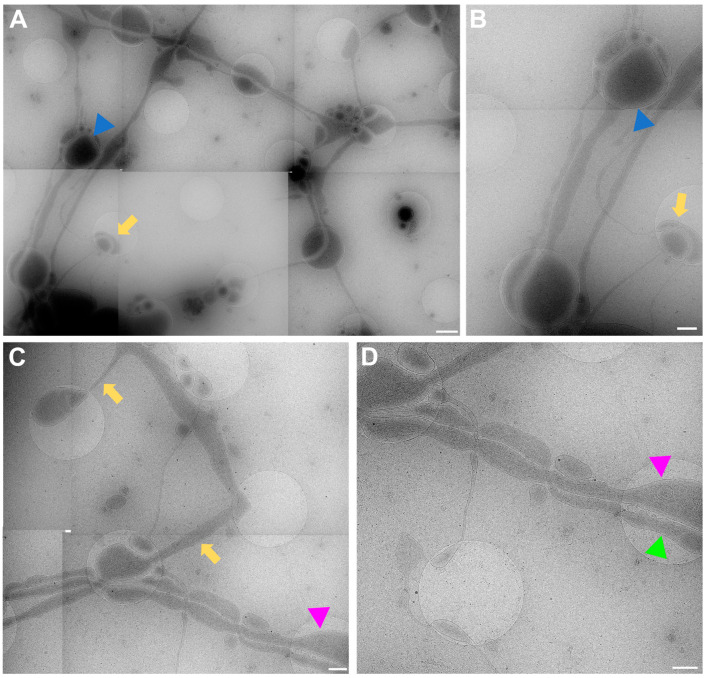
Cell process migration through and towards holes at lower (**A**,**C**) and higher (**B**,**D**) zoom, respectively. (**A**) Low-magnification view for (**B**), showing many neuronal processes migrating through holes, establishing synapses (blue triangles), and establishing potential new connections (yellow arrows). Corresponding structures marked with arrow and triangle in (**A**,**B**). (**C**) Migration of processes into holes (yellow arrows). Pink triangle indicates same structure in (**C**,**D**) for reference. (**D**) Neuronal processes bulge (pink triangle) or do not bulge (green triangle), depending on filling state. Scale bar (**A**) = 1 µm. Scale bars (**B**–**D**) = 500 nm. Images acquired at eBIC by IK.

**Figure 7:**
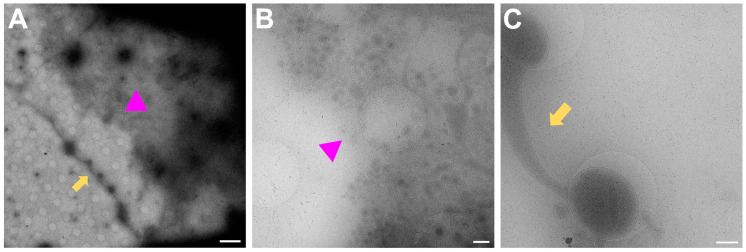
Regions to avoid for acquisition of images of synapses. (**A**) Gridsquare overview for (**B**) (pink triangle regions) and (**C**) (yellow arrows). Scale bar = 5 µm. (**B**) Search magnification view of the pink triangle region with few processes spreading from the top right corner and vesicles, some of which are being pinched off from the cell boundary at the top right corner. Scale bar = 500 nm. (**C**) Single neuronal process with mitochondria and synaptic vesicles filling the bulging in the holes. Scale bar = 500 nm. Images acquired at eBIC by IK.

The following figures (Figure 2, Figure 3, Figure 4, Figure 5, Figure 6 and Figure 7) will highlight the findings of the bespoke protocol and show what to expect at the different magnifications needed to set up a tomography session for the acquisition of functional synapses.

In Figure 2, an overview of the whole grid is shown at very low magnification (145×). This magnification is sufficient to determine if the neurons have grown well on the grid. Ideally, there should be many grid squares with distinct neuronal processes visible, as shown in Figure 2 (examples displayed with yellow arrows) and the fewer regions of densely packed neurons (red circles, Figure 2), the better, as the electron beam is not able to penetrate these regions.

In Figure 3, a magnification to cover one gridsquare was chosen to check ice thickness and sample quality. Synapses can mainly be found either on processes growing along each other or at a crossing of two processes. At gridsquare magnification, the holey carbon foil becomes more clearly visible and consists of a regularly spaced pattern of holes and carbon film. Cell bodies are now clearly visible as electron-dense regions (Figure 3B,C, white asterisk), and neuronal processes can be seen growing along the gridsquare (some examples marked with yellow arrows in Figure 3).

In Figure 4, the magnification used has to be sufficient to identify synapses for setup of tomogram positions. Figure 4A,B predate the time when search maps existed in Tomography (TFS, Tomo 4), so only single search magnification images were acquired. In Figure 4C,D, a stitched image of a search map is shown. All images show neuronal processes (yellow arrows in Figure 4) growing over the holey carbon support film with the blue arrow in Figure 4D pointing to a filled foilhole in contrast to the blue arrow in Figure 3A pointing towards an empty hole.

In Figure 5, lower (Figure 5A,C,E) and higher magnification (Figure 5B,D,F) images of 3 exemplary functional synapses are depicted. Figure 5B,D shows synapses where no fusion events could be detected, meaning the active zone plasma membrane (AZPM) is smooth, and synaptic vesicles are tethered to the AZPM, ready to be released upon action potential triggering [11,12]. On the other hand, in Figure 5F, fusion and endocytosis events could be observed as previously shown [12] (see also Supplementary Video S2), indicating activity at the time of freezing and thus providing documentation for functionality of the neurons grown. Mature synapses, consisting of a pre- and a postsynapse, can be found when two processes get close to each other and form a connection with each other. The presynapse is filled with synaptic vesicles, and vesicles can be seen tethered to the AZPM, ready to be released when triggered by an action potential. Tightly connected to the presynapse via the synaptic cleft is the postsynapse, which can easily be recognized as a much emptier space than the presynapse and via the dense projection lining the membrane opposite to the AZPM.

The data acquired by both experimenters independently very clearly show how the neuronal processes grow through and towards the holes in the carbon foil (Figure 4 and Figure 6). In Figure 6, it is especially clear how processes make detours to reach the holes (yellow arrows in Figure 6) and make detours to grow towards each other (yellow arrow in Figure 6B). Additionally, when zooming in on Figure 4, Figure 5, Figure 6 and Figure 7, it becomes clear that where the cell finds a suitable environment, be it over a hole or on carbon, the neuronal process expands/bulges out to accumulate material such as mitochondria and to establish synapses with a neighboring process.

In Figure 7, examples of regions that are unlikely to have mature synapses are shown. Figure 7A is the overview for both Figure 7B,C. In Figure 7B, many small vesicles are present, and some can be seen pinching off from the nearby cell upon zooming in. Figure 7C shows one single process which is filled with mitochondria and synaptic vesicles but since there is no other process nearby to form a connection with, a mature synapse will not be formed here.

## 3. Discussion

Here, we introduce an optimized protocol to obtain a primary neuron culture grown on EM grids that exhibit a good cell density for optimal growth for neurons to grow towards each other, establish connections, and grow functional synapses consisting of a pre- and a postsynapse in a thin environment ideally suited for direct imaging via cryo-ET with a measured thickness of about 200–300 nm. As proof of principle, the method has been performed by two of the authors at two different facilities, respectively, with comparable results.

Astrocytes are essential for a healthy neuron culture and promote neuron survival, synapse formation, and plasticity [21]. Therefore, astrocytes were used as a base layer on the 12-well plates. Initially, to avoid contact between astrocytes and neurons grown on the EM grid, the well bottom was elevated with a soldering iron similar to [14]. However, this proved to be unnecessary because once the astrocyte medium was replaced with neuronal medium, astrocyte growth came to a halt. With the astrocyte growth halted by the medium and the neurons seeded directly on the EM grid, astrocyte and neuron mixing on the grid was prevented, thus keeping both physically separated but close enough to exert the positive effects of a co-culture on neuron maturation.

Astrocytes were prepared as described in the method section and, following their 10-day growth period, were triturated and counted to a concentration between 50,000–300,000 cells/mL, then diluted to a total volume of 2 mL in DMEM. Best results were obtained using 100,000–200,000 cells/mL. After astrocytes were plated into 12-well plates, they were left to grow for 2 days, during which time it was critical to leave the plates as undisturbed as possible. Various coating conditions were tested, including fibronectin (FN), poly-D-lysine (PDL), UV irradiation, carbon coating, glass coverslips, and no coating at all. The best and simplest solution was not to coat the sterile plates at all, since astrocytes attached and grew very well.

Equally, EM grids were pre-treated with FN, PDL, carbon coating, or only flame sterilization as described in [6], and neurons were found to grow very well just on flame-sterilized grids. Flame sterilization will make the grids hydrophilic and sterile, and the grids can then either be placed directly into the sterile 12-well plates (as carried out by IK) or back into a grid box for a 20-min incubation period under UV light in a cell culture hood (as carried out by JR). The flame sterilization should be completed just prior to the incubation of neurons on the grid to maintain hydrophilicity.

The number of neurons (cells/mL) varied because one was a knockout mouse line and the other one an outbred mouse line, with outbred neurons being more viable, especially compared to the expression of a mutation in *SNAP25* knockout neurons that disinhibits spontaneous release (4K, [12,22]). Hence, a lower number of cells was used from the outbred mouse line to avoid overcrowding of the grid. However, while 250,000 cells/mL for the knockout neurons was found to be a good condition, in the same batch, some grids would be either scarcely populated or overgrown with neurons (which is to be expected when working with primary neuron cultures and has been observed previously [14]). For a new experimental setup trialling a range of cells/mL, it would be recommended to adjust for different experimenters as well as different mouse lines.

Importantly, in this protocol, neurons had been incubated on the bare flame-sterilized EM grid by adding a droplet of neuron suspension directly onto the grid, just enough to cover the grid. This first of all allows for the use of very small numbers of neurons (20 µL) compared to filling the bottom of a 12-well plate with neurons. At the same time, in our hands, we found this to give more reproducible results, since neurons will be confined to the EM grid upon attachment and not distribute freely in the whole well. A similar procedure has been described before with a droplet of 250,000 neurons/mL used to incubate directly on the EM grid [8].

Conveniently, because grid bars offer more support than the holey carbon film, neurons preferably attach to the grid bars, and their processes then grow towards each other and over the carbon support film. This behaviour is ideal since, ultimately, neurons need to find a responsive partner for a functional synapse to be formed [23]. Mainly thin processes grow over the gridsquare, and the cell bodies are mostly located on the grid bars. Consequently, the ice will be thin enough for direct imaging. Another interesting finding is that with this method of neuron preparation, the cells did not show signs of growing preferably on carbon but rather appeared to go straight through the holes and towards the next hole (Figure 5). One hypothesis is that since the astrocytes were sitting below the grid, perhaps communication and nutrient access are easier inside the holes. On the other hand, since neurons are attracted to each other, neurons will grow towards each other as opposed to growing towards astrocytes.

Another important point to mention is that different grid types were used by the two experimenters. Experimenter 1 used R2/1 grids, while experimenter 2 used R2/2 grids. Both proved to be comparable in terms of neuron and synapse growth as well as neuronal distribution. However, considering that neurons grown with this method do not avoid growing through holes, the R2/1 grids should be the preferred grid choice, since there will be more holes to collect data from.

It is also important to consider the duration of neuron growth, since from DIV 14 onwards more synapses can be found. Until DIV11, there are almost no functional synapses, from day 12 more can be found, and day 14 is the threshold for neurons grown without a medium change and having a good number of synapses. Since medium changes can perturb the neurons, the ideal freezing time was found to be at DIV14. Thereafter, with the medium becoming old, more neurons can be found that possess large accumulations of black dots inside the mitochondria, which have been characterized as calcium/phosphate accumulations [24,25]. In neurons, this could potentially indicate that those accumulations are a sign of age and stress [26] in cells rather than a typical feature, as they are rarely observed in young, healthy neuron cultures (mitochondria visible in Figure 4, Figure 5 and Figure 6) and in synaptosome preparations [11,12] or in Epon embedded mouse cerebellum sections [27].

Once the neurons are ready for vitrification, picking up the grids from 12-well plates poses a significant challenge if they have been treated well and are lying flat on the well-plate. One option, if material is not a concern, is to use 6-well plates instead of 12-well plates. In 6-well plates, the angle of the tweezer can be much shallower, which makes picking the grid up significantly easier.

Taken together, this protocol describes a method to reliably grow primary neurons which made functional synapses, on EM grids, thin enough to be imaged directly on a Titan Krios without prior milling. This was achieved by introducing various changes to existing protocols in the field, introduced in the introduction, in short, (1) the use of astrocytes appears to be beneficial for synapse formation and neuron growth over foilholes, (2) the use of PDL has been omitted, thus saving preparation steps, and (3) neurons are incubated as droplets on the EM grid, saving material and providing a more controlled environment. A future experiment to prolong neuron growth by up to 2 weeks and potentially obtain more functional synapses could be to add 1 mL of neuronal medium once a week to the 2 mL of existing growth medium. This would avoid disturbing the neurons too much, would add some fresh medium, and at the same time retain the old medium with the existing nutrients.

Being able to more reliably find functional synapses for studies via cryo-ET is important when studying synapse morphology in a close-to-native state with high structural resolution. Understanding the structures in a healthy, resting state will provide the basis for studying time-resolved processes such as synaptic vesicle exo- and endocytosis but also for investigating disease models in neurodegeneration and neurotoxicity where the synapse is affected [28,29,30].

## 4. Materials and Methods

### 4.1. Experimental Setup

Primary neurons were grown under different culturing conditions in order to establish a protocol that would provide growth of mature neurons with functional synapses, consisting of a pre- and a postsynapse, for direct imaging via cryo-ET. The method described in this section will focus on the method that was found to work both for outbred mice (experiments conducted at the electron Bio-Imaging Centre (eBIC) at Diamond Light Source by IK) as well as for the much more susceptible SNAP-25 knockout mouse line used in our recent research paper [12] (developed at Copenhagen University by JR). 

While ref. [12] addressed the results that can be achieved from a neuroscientific perspective, here we will focus on the detailed description of the method to make it accessible to a wide community.

### 4.2. Animals

SNAP-25 KO C57BL/6J mice (B6.129X1-Snap25^tm1Mcw^/J): Heterozygous animals were routinely backcrossed to C57BL/6J to generate new heterozygotes. The strain was kept in a heterozygous condition and timed heterozygous crosses and caesarean section were used to recover knockout embryos at embryonic day 18 (E18). Pregnant females were culled via cervical dislocation; embryos of either sex were collected and culled via decapitation. Permission to keep and breed SNAP-25 (Snap25^tm1Mcw^) mice was obtained from the Danish Animal Experiments Inspectorate (2018-15-0202-00157, approved on 28 August 2018) and followed institutional guidelines as overseen by the Institutional Animal Care and Use Committee (IACUC). CD1 outbred mice stock (ICR/H[CD1]): these were used to create astrocytic cultures. Newborns (P0-P2) of either sex were used. Pups were culled via decapitation. For the second set of experiments, the ICR/H[CD1] outbred strain was used to create astrocytic and neuronal cultures. Newborns (P0–P2) of either sex were used. Pups were culled via decapitation. These mice were bred at the Mary Lyon Centre, Harwell, and were housed in individually ventilated cages in a specific pathogen-free environment. These mice were kept under controlled light (light 7 a.m. to 7 p.m., dark 7 p.m. to 7 a.m.), temperature (21 °C ± 2 °C), and humidity (55% ± 10%) conditions. They had free access to water (25 p.p.m. chlorine) and were fed ad libitum on a commercial diet (SDS Rat and Mouse No.3 Breeding diet (RM3)).

### 4.3. Preparation of Astrocytic and Neuronal Culture

Parts of the procedure have been published before [12,22]. However, important changes have been made to grow primary neurons on EM grids with very low amounts of material and in order to keep the flow of the paper, previously published method sections were integrated into newly written sections. The preparation details for the solutions used in the following steps can be found in Appendix A Table A1. Substance resource information can be found in the Supplementary Table S1.

**Figure 8:**
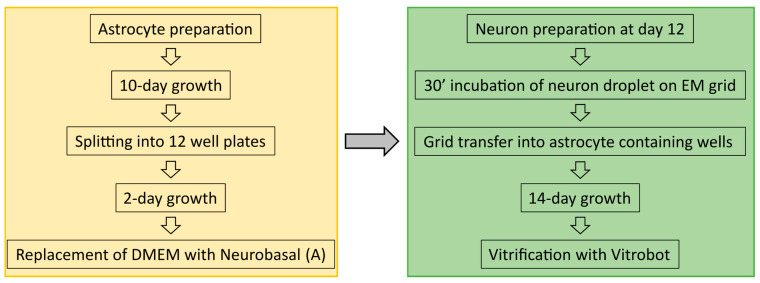
Overview off the sample preparation timeline from astrocyte culturing to neuron preparation and vitrification.

Astrocytes were isolated from CD1 outbred mice (P0–P2). After decapitation, heads were placed in HBSS-HEPES medium. The cortices were isolated from the brains, and the meninges were removed (dura, pia, and arachnoid mater). The cortices were chopped into smaller fragments and transferred to a tube containing 0.25% trypsin dissolved in Dulbecco’s MEM (DMEM) supplemented with 10% Foetal calf serum, 20,000 IU Penicillin, 20 mg Streptomycin and 1% MEM non-essential Amino Acids. Fragments were incubated for 15 min at 37 °C. Subsequently, inactivation medium (12.5 mg Albumin + 12.5 mg Trypsin-Inhibitor in 10% DMEM) was added and the tissue washed with HBSS-HEPES. Tissue was triturated until a smooth cloudy suspension appeared. Cells were plated in 80 cm^2^ flasks with pre-warmed DMEM, one hemisphere per flask, and stored at 37 °C with 5% CO_2_. Glial cells were ready to be used after 10 days. Glial cells were washed with pre-warmed HBSS-HEPES. Trypsin was added, and the flasks were incubated at 37 °C for 10 min. Cells were triturated and counted with a Burker chamber before plating 100,000 cells/mL into untreated 12-well plates containing DMEM to a total volume of 2 mL of solution per well. For a further two days, the astrocytes would reattach and continue to grow before neurons were added following the preparation described below. An overview into astrocyte and neuron preparation timelines is shown in Figure 8.

Hippocampal neurons were isolated from either E18 SNAP-25 KO or WT mice, or P0-P1 CD1 mice. The SNAP-25 KO pups were selected based on the absence of motion after tactile stimulation and bloated neck [31]; the genotype was confirmed via PCR in all cases. The pups were culled via decapitation, and heads were put in HBSS-HEPES medium. The cortices were isolated from the brains, and the meninges were removed. The hippocampi were cut from the cortices before being transferred to a tube containing 0.25% trypsin dissolved in HBSS-HEPES solution. Fragments were incubated for 20 min at 37 °C. Afterwards, the tissue was washed with HBSS-HEPES. The hippocampi were triturated, and the cell count was determined with a Burker chamber.

### 4.4. Sample Preparation

A total of 200 mesh gold–carbon R2/2 and R2/1 EM grids (Quantifoil, Großlöbichau, Germany) were flame-sterilized as previously described in [6], and one grid per well was placed into empty sterile 12-well plates. The flame-sterilized grids can be kept in any sterile environment; here 12-well plates were chosen to avoid additional transfers and allow for individual incubation of each grid with the neuron suspension as described next. A droplet of 20 µL of solution containing 250,000 cells/mL for the knockout mouse line and 12,000 cells/mL for the outbred mice were pipetted onto the flame-sterilized EM grids. Neurons were then incubated for 30 min at 37 °C in an incubator at a 5% CO_2_ concentration. During the incubation of the neurons, the astrocyte growth medium, in the 12-well plates, was replaced with 2 mL prewarmed NB medium (Neurobasal with 2% B-27, 1 M HEPES, 0.26% Glutamax, 14.3 mM β-mercaptoethanol, 20,000 IU Penicillin, 20 mg Streptomycin) for the E18 pups or 2 mL prewarmed NB-A medium (Neurobasal-A with 2% B-27, 1% Glutamax, 20,000 IU Penicillin, 20 mg Streptomycin) for the P0-P1. After 30 min, the grid containing the lightly attached neurons was carefully transferred into the 12-well plate containing the astrocytes using sharp ceramic tweezers (#0208-5HDC/B-CO-1 Dumont, Hatfield, PA, USA). The 12-well plate was then incubated for further 14 days at 37 °C in an incubator with 5% CO_2_ concentration. During this time minimal disturbance is required and a medium change is not necessary.

After 14 days, grids (both at the CFIM and at eBIC) were plunge frozen with a Vitrobot (Thermo Fisher Scientific (TFS), MARK IV, Waltham, MA, USA) with a blot time of 3 s and a blot force of −10, wait time and drain time were not used, humidity was set to 100% at 4 °C. 4 µL undiluted 10 nm BSA gold tracer (#210.033, AURION Immuno Gold Reagents & Accessories, Wageningen, The Netherlands) was added directly onto the grid prior to plunge freezing. Grids were directly vitrified in their growth medium with the added BSA gold tracer.

### 4.5. Data Collection and Analysis

For the first set of data obtained at the CFIM at the University of Copenhagen, tomograms were acquired at a Titan Krios G2, equipped with a Falcon 3 direct electron detector (TFS) without energy filter. The Falcon camera was operated in linear mode, no frames were collected, and CTF correction was applied during reconstruction in IMOD. Tilt series were acquired using the Tomography software (TFS, Tomo version 4) for automated acquisition recorded typically from −60° to 60° with a 2° angular increment and an unbinned pixel size of 0.37 nm. Defocus was set between −6 to −10 µm, and the total electron dose used was about 80–100 e^−^/Å^2^. The tomograms were acquired from 4 grids (3 KO and 1 WT sample) with a total of 19 functional synapses having been acquired.

The second set of data was collected at the Titan Krios G2 at eBIC, equipped with a Falcon 4i direct electron detector (TFS) and the SelectrisX energy filter. The Falcon camera was operated in counting mode, 6 frames per tilt were collected and motion corrected by motioncor2 [32], followed by CTF estimation [33]. Tilt series were acquired using the Tomography software (TFS, Tomo version 5.8–5.10) for automated acquisition recorded with a dose symmetric scheme starting at 0° with a tilt span of 60° with a 3° angular increment and an unbinned pixel size of 0.3 nm. Defocus was set between –2.5 to –5 µm and the total electron dose used was about 120 e^−^/Å^2^. The tomograms were acquired from 2 grids with a total of 11 functional synapses having been acquired.

3D reconstruction was done in IMOD [34]. The alignments were done using the automated fiducial tracking function and the 3D reconstructions were done using the weighted back projection followed by a nonlinear anisotropic diffusion (NAD) filtering.

## Data Availability

Data are contained within this article and the corresponding supplementary material. Additionally, if required, the data presented in this study are available on request from the corresponding author.

## Supplementary Materials

The supporting information can be downloaded at: https://www.mdpi.com/article/10.3390/ijms242015191/s1.

## Appendix A

**Table A1:** Media preparation. Each solution is prepared in order from top to bottom.

DMEM (astrocyte growth medium)
450 mL DMEM (remove 50 mL DMEM—keep for dissolving the trypsin inhibitors)
50 mL FBS
1000 µL Penicillin/Streptomycin (20,000 IU Penicillin and 20 mg Streptomycin)
5 mL MEM-Non-Essential Amino Acids
Mix together in the DMEM bottle
Filter sterilize
HEPES—make 1 molar stock (molecular weight = 238.31)
Weight out 5.96 g of HEPES and dissolve it in 25 mL of dH2O
Aliquot in 3.5 mL and freeze
HBSS-HEPES
496.5 mL HBSS (remove 3.5 mL of HBSS and dispose of it)
Add 3.5 mL 1M HEPES
Mix in the HBSS bottle
Adjust the pH with NaOH to 7.35
Filter sterilize
Neurobasal-A (neuronal preparation from P0/P1 pups)
500 mL Neurobasal-A
Add 5 mL Glutamax I
Add 1 mL Penicillin/Streptomycin
Mix in the Neurobasal-A bottle
Filter sterilize
Add 10 mL B27 Supplement
