# Supplementary material for: An Improved Method for Growing Primary Neurons on Electron Microscopy Grids Co-Cultured with Astrocytes
Source: Int J Mol Sci. 2023 Oct 14;24(20):15191. doi: 10.3390/ijms242015191 (PMC10606997; doi:10.3390/ijms242015191)
Supplement: Supplementary file 1 [file ijms-24-15191-s001.zip › Supplementary Information.pdf]

Supplementary Information

Table S1. Substance resources.

Substance	Supplier & Order number	Comments
**MEM-Non-Essential Amino Acids**	Invitrogen 11140035	
**DMEM**	Invitrogen 31966047	
**Penicillin/Streptomycin**	Invitrogen 15140122	
**Fetal Bovine Serum (heat inactivated)**	Invitrogen 10500064	thaw, aliquot in 50 mL, freeze
**Trypsin, 2.5% (10x)**	Gibco 15090046	
**Trypsin Inhibitor (powder)**	Sigma-Aldrich T-9253	
**Bovine serum albumin (powder)**	Sigma-Aldrich A9418 – 10 g	
**0.05% Trypsin/EDTA**	Gibco 25300-054	
**HBSS**	Invitrogen 24020133	
**Neurobasal**	Invitrogen 21103049	
**B27 – Supplement**	Invitrogen 17504044	
**Glutamax-I Supplement**	Invitrogen 35050038	
**HEPES (powder dissolved in miliQ water)**	Sigma-Aldrich H4034	freeze 3.5 ml aliquots

Video S1: Tomographic reconstruction of a mature synapse (video corresponding to Figure 5D).

Video S2: Synaptic vesicle fusion and endocytosis in a mature synapse (video corresponding to Figure 5F).
